# Phase 2 trial of hypoxia activated evofosfamide (TH302) for treatment of recurrent bevacizumab-refractory glioblastoma

**DOI:** 10.1038/s41598-021-81841-0

**Published:** 2021-01-27

**Authors:** Andrew J. Brenner, John Floyd, Lisa Fichtel, Joel Michalek, Kunal P. Kanakia, Shiliang Huang, David Reardon, Patrick Y. Wen, Eudocia Quant Lee

**Affiliations:** 1 Mays Cancer Center (A.J.B.), The University of Texas Health Science Center at San Antonio, 7703 Floyd Curl Drive, San Antonio, Texas 78229-3900 USA; 2 South Texas Oncology and Hematology, San Antonio, TX USA; 3 Dana Farber Cancer Institute, Boston, MA USA

**Keywords:** Chemotherapy, Drug development, Targeted therapies, CNS cancer

## Abstract

Evofosfamide (Evo or TH302) is a hypoxia-activated prodrug which is reduced leading to the release of alkylating agent bromo-isophosphoramide mustard, which has shown safety and signals of efficacy in a prior phase 1 study in recurrent glioblastoma. We performed a dual center single-arm Phase II study to expand on the safety and efficacy of Evo plus bevacizumab in bevacizumab refractory glioblastoma. 33 patients with bevacizumab refractory GBM received Evo 670 mg/m^2^ in combination with Bevacizumab 10 mg/kg IV every 2 weeks. Assessments included adverse events, response, and survival. Median age of patients was 47 (range 19–76) and 24 (69%) were male. At the time of study entry, 9 (26%) had ongoing corticosteroid use. ECOG performance status was 0 or 1 in 83% of patients. Patients were mostly heavily pretreated with 77% have three or more prior regimens. A total of 12 patients (36%) suffered grade 3–4 drug associated adverse event (AE); no grade 5 AE were reported. Of the 33 evaluable patients, best response was PR in 3 (9%), SD in 14 (43%), and PD in 16 (48%) with responses confirmed by a second reviewer. Median time to progression of disease was 53 days (95% CI 42–113) and Median time to death was 129 days (95% CI 86–199 days). Progression free survival at 4 months (PFS-4) on Evo-Bev was 31%, which was a statistically significant improvement over the historical rate of 3%. The median overall survival of patients receiving Evo-Bevacizumab was 4.6 months (95% CI 2.9–6.6). The progression free survival of patients on Evo-Bevacizumab met the primary endpoint of progression free survival at 4 months of 31%, although the clinical significance of this may be limited. Given the patient population and Phase II design, these clinical outcomes will need further validation.

## Introduction

Glioblastoma (GBM, Grade IV astrocytoma) is the most common primary malignant brain tumors in adults. Approximately 13,000 cases of GBM will be diagnosed each year in the United States, and it remains incurable with a median survival below 2 years^1^. Temozolomide, radiation and tumor treatment fields constitute Food and Drug Administration (FDA) approved first line therapy options for GBM^2,3^. For recurrent disease, while a number of salvage treatment options exist, none have improved overall survival and hence additional treatment options are needed.

GBM is a rapidly proliferating neoplasm with a supply–demand mismatch leading to an environmental deficiency of oxygen^4–6^. Despite vascular proliferation, the ensuing hypoxia ultimately leads to the formation of necrotic areas, a pathognomonic histologic feature of GBM^7^. The volume and intensity of hypoxia in GBM are strongly associated with a shorter time to progression and poorer survival^8^.

While bevacizumab has become a standard part of salvage therapy for recurrent GBM, multiple studies have shown it does not improve survival^9–12^. Increasing evidence points to the root cause of angiogenesis, hypoxia, as a driving force for resistance to Bevacizumab^13^. This is supported by biomarker studies in which surrogates of hypoxia such as carbonic anhydrase (CAIX), hypoxia-inducible factor 1α (HIF-1α), hypoxia-inducible factor 2α (HIF-2α), or stromal derived factor 1α are increased at the time of progression after initially responding to Bevacizumab^14,15^, or following progression with the VEGFR inhibitor cedirinib^16^.

Evofosfamide (Evo or TH 302), is a nitroimidazole prodrug of the cytotoxin, bromo-isophosphoramide mustard (Br-IPM) and considered a second generation Hypoxia Activated Pro-drug (HAP) with benefit compared to first generation HAP’s including its ability to diffuse into hypoxic regions without activation by DT diaphorase. Evo is activated preferentially in hypoxic conditions; therefore, it is unlikely to be present in nonpathologic tissues in the body^17^. When exposed to hypoxic conditions, Evo is reduced by intracellular reductases leading to the release of the alkylating agent Br-IPM. Br-IPM can then act as a DNA crosslinking agent, also diffusing to adjacent cells in normoxic regions and thus act as a cytotoxic agent outside of the hypoxic activation zone. Given the hypoxic nature of GBM, and the tendency of antiangiogenics to exacerbate tumoral hypoxia, we hypothesized that Evo may be active in recurrent glioblastoma following bevacizumab failure. Our prior Phase 1 study showed the combination to be safe and provided preliminary evidence of potential efficacy^18^ with a 17% response rate and 61% clinical benefit rate. In order to further evaluate the activity and safety of Evo in bevacizumab refractory GBM, we undertook an open label, single-arm, dual center, Phase II study of Evo with Bev after Bevacizumab failure.

## Methods

### Patients

Eligible patients were adults of 18 years or greater with progressive or recurrent glioblastoma and radiographic evidence of progression following bevacizumab. MRI demonstrating progression prior to study consideration was defined by Radiographic Assessment in Neuro-Oncology (RANO) criteria^19^. Patients were required to have received prior radiation therapy and temozolomide chemotherapy, as well as bevacizumab. Patients were required to have an Eastern Cooperative Group (ECOG) performance status of 2 or less, with adequate hematologic, renal, and liver function. Exclusion criteria included receiving warfarin or enzyme-inducing anti-epileptic agents within 14 days of study drug, evidence of acute intracranial or intratumoral hemorrhage, ongoing toxicity from prior therapy of grade 2 or more, or contraindications for continued therapy with bevacizumab (which include wound dehiscence, uncontrolled hypertension, or serious intercurrent illness). The protocol was approved by the institutional review board at the respective institutions (University of Texas Health Science Center at San Antonio and Dana Farber Cancer Institute), and all patients provided written informed consent. All methods were carried out in accordance with Good Clinical Practice and in accordance with local guidelines and regulations. This trial was registered with www.clinicaltrials.gov (NCT02342379) on 19 Jan 2015.

### Study design

This phase II, open label, single-arm, study evaluated evofosfamide in combination with bevacizumab following bevacizumab failure. Patients received evofosfamide at 670 mg/m^2^ and bevacizumab 10 mg/kg, given concurrently intravenously every 2 weeks until disease progression or unacceptable toxicity. The primary endpoint was progression free survival at 4 months (PFS4) with comparison to historical control^20^. Secondary endpoints were safety and overall survival.

### Dose modifications and toxicity management

Dose modifications for toxicity, particularly hematologic and skin toxicity, were assessed independently at each visit and managed as previously described^18^. Briefly, dose reductions were not required for toxicity less than grade 3 with the exception of grade 2 skin toxicity, which required a dose reduction of 25% upon resolution to grade 1. For non-hematologic toxicity of grade 3 (other than ALT/AST elevation, nausea or vomiting), a 25% dose reduction was required. For any non-hematologic grade 4 adverse events treatment was discontinued. Reduced absolute neutrophil count (ANC) of 1000–1499, and platelet counts of 50,000–75,000 were managed with a 25% dose reduction. Lower ANC or platelets required doses to be held until recovery to 1500 and 100,000, respectively. Hemoglobin was required to be ≥ 9 g/dL at Cycle 1/Day 1 and ≥ 8 g/dL for all subsequent doses. All patients were advised to use Preparation H cream (pramoxine/phenylephrine/glycerin/petrolatum) immediately prior to infusions to prevent perineal rash and anal mucositis. If rash or anal mucositis reached grade 1, Silvadene 1% cream and triamcinolone 0.1% cream both applied twice daily was added to the skin care regimen. Patients were provided skin care medications and instruction prior to starting treatment.

### Radiographic imaging and response

MRI scans were performed on 3 T MRI scanners (Philips, GE, and Siemens). Each scanning session consisted of 3D pre- and post-contrast T1 weighted images, FLAIR (fluid-attenuated inversion recovery), diffusion weighted images, and Dynamic Susceptibility Contrast (DSC). T1 pre-contrast, fluid attenuation inversion recapture (FLAIR) images were acquired before contrast injection. After the first intravenous injection of 0.1 mmol/kg of a standard gadolinium-based contrast agent, dynamic contrast enhancement (DCE) and diffusion weighted images (DWI) were acquired. For DCE, injection took place after 10 baseline frames were obtained. The second injection was for DSC perfusion MRI and T1 post contrast images. All assessments were performed per RANO criteria.

### Statistical methods

This was a single-arm Phase II study using a two-stage Simon design with regard to the proportion progression free at four months. Based on historical data^20^ we estimated a poor proportion progression free at four months as 0.109 and a good proportion as 0.289. With these parameter estimates, α = 0.05, and 80% power, the optimal two-stage Simon procedure specified 11 subjects in the first stage and study termination if 1 or fewer subjects in the first stage were progression-free at 4 months. If the trial went to the second stage, then a total of 33 subjects would be studied, and if the total number progression-free at 4 months among the 33 was less than or equal to 6 then the test drug was to be rejected. Patients deemed unevaluable due to withdrawal or noncompliance before completing the first cycle without radiographic or clinical evidence of progression were replaced. Overall survival was defined as the interval from the start of treatment until death. Continuously distributed data are summarized with the mean plus or minus one standard deviation and categorical outcomes described with frequencies and percentages.

## Results

### Patient characteristics

A total of 41 patients were enrolled from June of 2015 through June of 2017 with 19 at Dana Farber Cancer Institute and 22 at UT Health San Antonio. Of those 41 patients consented, 6 patients failed screening with one due to abnormal transaminases, one due to thrombocytopenia, and four due to poor performance status. Of the 35 patients receiving treatment, the median age was 47 (range 19–76) and 24 (69%) were male (Table 1). At the time of study entry, 9 (26%) had ongoing corticosteroid use. ECOG performance status was 0 or 1 in 83% of patients. Two of the 35 withdrew consent prior to evaluation and were excluded. Given many patients were referred from community-based practices prior to publishing of the updated WHO criteria^21^, molecular marker were only available on a subset. MGMT status was methylated in 26%, unmethylated in 32%, and unknown in 42%. IDH mutations were identified in 29%, not seen in 46%, and unknown in 25%. Patients were mostly heavily pretreated with only 8 (23%) having received two prior regimens, with 19 (54%) three prior regimens, 5 (14%) four prior regimens, and 3 (9%) receiving 5 to 7 regimens before enrolling on study. All patients received at least two therapeutic agents before receiving the bevacizumab plus evo combination, with 15 (43%) receiving 3 agents, 8 (23%) receiving 4 agents, and 4 (11%) receiving between 5 and 8 agents prior to enrollment. All had progressed on at least one prior bevacizumab regimen, with 8 (23%) having progressed on two prior bevacizumab regimens. The median time to progression on prior bevacizumab therapy before study entry was 105 days. Demographics did not vary significantly with site (all p > 0.05).

**Table 1:** Table illustrating patient characteristics.

Patient characteristics	All patients (n = 35)
**Age**	
Median age	47 years
Range	19–76 years
Gender: male	24 (69%)
Patients with ongoing corticosteroid therapy	9 (26%)
ECOG performance status (0 to 1)	29 (83%)
**Pretreatment status (number of prior regimens)**^**a**^	
2	8 (23%)
3	19 (54%)
More than 3	8 (23%)
**Pretreatment status (number of prior agents used)**^**b**^	
3	15 (43%)
More than 3	12 (34%)
**IDH status**	
Unmutated	16 (46%)
Mutated	10 (29%)
Unknown	9 (25%)

^a^All patients had progressed on at least one prior bevacizumab regimen, with 8 (23%) having progressed on two prior bevacizumab regimens. The median time to progression on prior bevacizumab therapy before study entry was 105 days.

^b^All patients had at least 3 prior agents used before enrollment in the trial.

### Safety

The combination of Evo with bevacizumab was well overall well tolerated. One grade 4 drug associated adverse event (AE) of small bowel perforation felt to be likely related to bevacizumab occurred. Eleven grade 3 AEs occurred during the course of the study, including 5 of mucositis, 2 of pain secondary to mucositis, 2 thrombocytopenia, 1 perineal skin excoriation, and 1 fatigue. The most common AE of any grade was mucositis which occurred in 15 subjects (43%) and was most commonly anal or peri-anal in 73% of cases and oral in 27%. Other common AEs included fatigue (34%), rash (26%), thrombocytopenia (20%), hyperpigmentation (17%), skin ulceration (17%) and neutropenia (11%). All other AEs occurred in less than 10% of subjects and are detailed in Table 2.

**Table 2:** Table displaying the incidence of adverse effects (AE) in patients receiving Evo 670 mg/m^2^ in combination with Bevacizumab 10 mg/kg IV every 2 weeks (n = 35).

Adverse effect	All grades incidence (%) (n = 35)	Number of patients with grade 3 AE or higher (%)
Mucositis	43	5 (14)
Fatigue	34	1 (3)
Rash	26	0 (0)
Thrombocytopenia	20	2 (6)
Nausea	17	0 (0)
Skin ulceration	17	1 (3)
Hyperpigmentation	17	0 (0)

Adverse effects with all grades incidence lower than 10% are not included in this table, Grade 2 Anemia had highest incidence among this group (2%) with no reported Grade 3 or higher Anemia.

### Tumor response, time to progression, and overall survival

**Figure 1:**
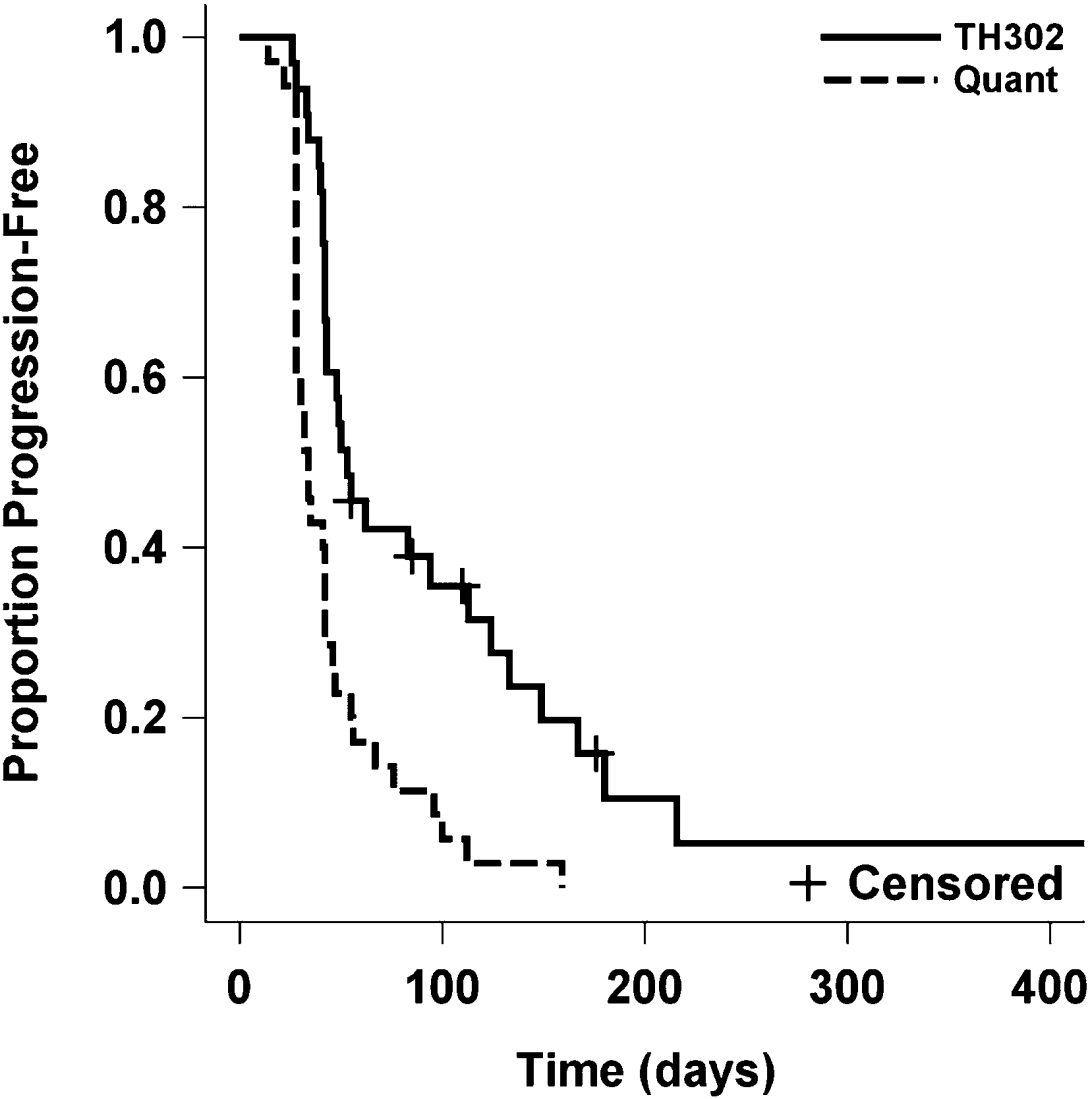
Kaplan–Meier estimate plots for PFS TH302 and Quant revealed p < 0.001 for PFS.

**Figure 2:**
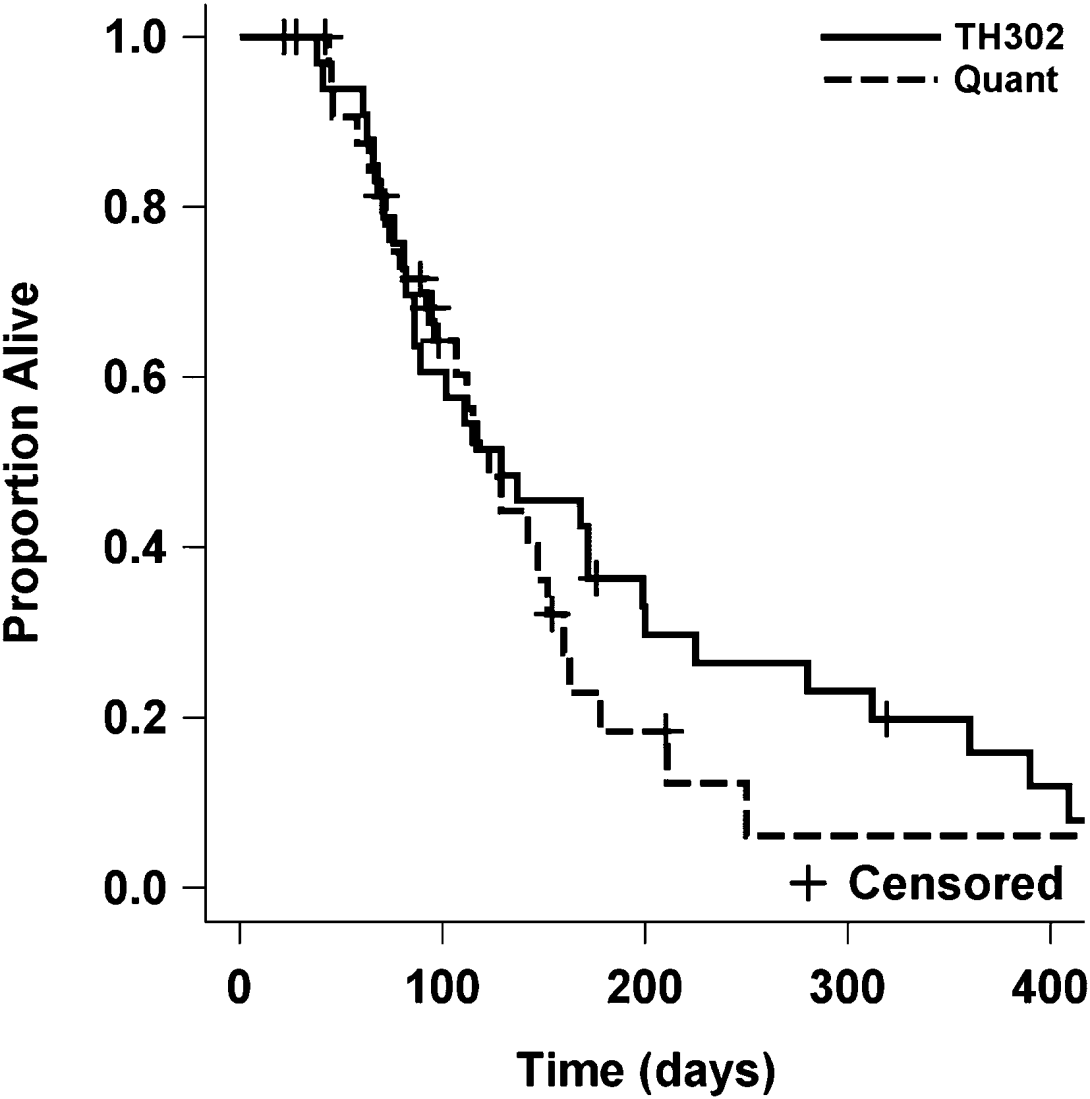
Kaplan–Meier estimate plots for OS comparing TH302 and Quant revealed p = 0.29 for OS.

Of the 33 evaluable patients, best response was PR in 3 (9%), SD in 14 (43%), and PD in 16 (48%) with responses confirmed by a second independent reviewer. PFS at 4 months was 31%, which met the primary endpoint. The median time to progression and death were 53 days (95% CI 42–113) and 129 days (95% CI 86–199 days), respectively. Progression free and overall survival Kaplan Meier curves are shown in Figs. 1 and 2, respectively. In order to assess potential impact of IDH mutation status, Kaplan Meier curves were generated for PFS and OS by IDH mutation status (detected vs not detected; supp materials Fig. S1). Patients with IDH mutation had a shorter median time to progression (42.5 vs 83 days [95% CI 33–94 and 29–133]) and death (94 vs 152.5 days [95% CI 38–172 and 86–280]), although the sample size was small and this did not reach significance. No correlation was observed between the timing from the last bevacizumab regimen and either PFS (HR 1.00, p = 0.66) or OS (HR 1.00, p = 0.61). Similarly, no correlation was observed between ECOG performance and PFS (HR 1.70, p = 0.13) or OS (HR 1.8, p = 0.13).

The Quant study^20^ was selected as a historical control and included 54 patients with recurrent malignant gliomas who progressed on a bevacizumab containing regimen and were then treated with an alternate bevacizumab-containing regimen. Kaplan–Meier estimate plots for PFS and OS comparing TH302 and Quant revealed p < 0.001 for PFS (Fig. 1) and p = 0.29 for OS (Fig. 2).

## Discussion

Once patients progress on Bev, survival remains very poor even if treated on a second Bevacizumab containing regimen with a historical OS of 3 months^20^. Additionally, radiographic responses are rare for Bevacizumab refractory disease^15,20,22–25^, and this is especially true following multiple bevacizumab regimens. In this phase II study, despite a heavily pretreated population including over half with three prior regimens and an additional quarter with 4 or more regimens, a statistically significant difference in progression free survival at 4 months and Kaplan–Meier estimate for progression was observed which suggests some activity of Evofosfamide in this difficult to treat population. The similarities between the Quant study and our study include the fact that the study population in both cases was heavily pre-treated with 70% of participants in the former study and 77% in the latter having received 3 or more prior chemotherapeutic regiments, also in both studies, the median age of the subjects was similar (47 in our study versus 50 in the Quant study). Also, in both studies there was no exclusion of participants based on number of episodes of progressive disease. The key difference in the study participants, however, is that our study only enrolled recurrent glioblastoma whereas the Quant study included all recurrent malignant gliomas of which only 65% of participants had glioblastoma. In addition, the majority of our study subjects had ECOG scores of 0–1, as compared to the Quant study subjects who’s median KPS score was 70 (which is equivalent to ECOG score of 2), and data was not available regarding IDH mutation status for the Quant study. We did find that patients with IDH mutation did not have a statistically significant difference in progression, and no correlation was present between outcome and performance status. Yet, these differences may have played a role in the progression free survival differences.

Following the design of this study, a retrospective pooled analysis by Reardon et al^26^ was published of patients receiving subsequent therapy following completion of one of five different bevacizumab clinical trials for recurrent GBM. Fifty six percent (n = 55) went on to receive a subsequent bevacizumab regimen. The median age of those subjects (52 years) in was similar to that of our study subjects (47 years). Median PFS on second Bev regimens was 2.8 months (95% CI 1.7–3.5) and PFS at 6 months was 15.6% (95% CI 7.3–26.6). No responses were noted. On comparing our study to the Reardon (2012) study, no statistical difference in PFS was observed. Key differences included participants in the pooled retrospective analysis had 3 or less prior regimes versus 66% of participants in our study, patients received a number of different bevacizumab regimens and dosage initially and on progression, no formal protocol defined evaluation for progression was specified, and both treatment and evaluation could occur either locally or at the study institution depending on patient preference. These differences with this pooled analysis could account for a lack of statistical difference in PFS between these studies and therefore should be taken with caution.

Median overall survival for Evo/Bevacizumab was favorable at 4.6 months and higher than the survival of approximately 3 months reported for patients receiving a second Bevacizumab containing regimen after Bevacizumab failure^20,23^. The Evo-Bevacizumab regimen appears to provide a limited increase in PFS and OS compared to previous regimens^17,18^ in recurrent glioblastoma with a comparable toxicity profile^17,18^. Given the limited patient population, challenging nature of studies in the Bev refractory setting, and phase II design these clinical outcomes will however need further validation. Additional analysis of hypoxic volume, perfusion, anatomic radiographic features, and metabolic features as predictors of benefit is to be reported separately.

## Supplementary Information

Supplementary Information
