# Supplementary material for: Phase 2 trial of hypoxia activated evofosfamide (TH302) for treatment of recurrent bevacizumab-refractory glioblastoma
Source: Sci Rep. 2021 Jan 27;11:2306. doi: 10.1038/s41598-021-81841-0 (PMC7841164; doi:10.1038/s41598-021-81841-0)
Supplement: Supplementary file 1 — Supplementary Information [file 41598_2021_81841_MOESM1_ESM.docx]

Title: Phase 2 Trial of Hypoxia Activated Evofosfamide (TH302) for Treatment of Recurrent Bevacizumab-Refractory Glioblastoma

Author’s Full Names: Andrew Brenner MD PhD^1,4^, John Floyd MD^1^, Lisa Fichtel MD^2^, Joel Michalek PhD^1^, Kunal P Kanakia MD^1^, Shiliang Huang^1^, David Reardon MD^3^, Patrick Y. Wen MD^3^, Eudocia Quant Lee MD^3^

Affiliation: ¹University of Texas Health San Antonio Cancer Center (A.J.B.), The University of Texas Health Science Center at San Antonio, 7703 Floyd Curl Drive, San Antonio, Texas 78229-3900

^2^South Texas Oncology and Hematology, San Antonio, TX

^3^Dana Farber Cancer Institute, Boston, MA

Running Title: Evofosfamide for Recurrent GBM

^4^Corresponding Author: Andrew J. Brenner, Telephone: 210-450-5936, Fax: 210-692-7502, Email Address: dr_brenner@yahoo.com

Supplementary Figure

**A. B.**

**Figure S1. Kaplan Meier curve showing PFS (A) and OS (B) for patients with EVO plus Bev in the IDH mutated and unmutated patients.**
